# Cryptanalysis and Improvement of a Biometric-Based Multi-Server Authentication and Key Agreement Scheme

**DOI:** 10.1371/journal.pone.0149173

**Published:** 2016-02-11

**Authors:** Chengqi Wang, Xiao Zhang, Zhiming Zheng

**Affiliations:** Key Laboratory of Mathematics, Informatics and Behavioral Semantics, Ministry of Education, and School of Mathematics and Systems Science, Beihang University, Beijing 100191, China; King Saud University, Kingdom of Saudi Arabia, SAUDI ARABIA

## Abstract

With the security requirements of networks, biometrics authenticated schemes which are applied in the multi-server environment come to be more crucial and widely deployed. In this paper, we propose a novel biometric-based multi-server authentication and key agreement scheme which is based on the cryptanalysis of Mishra et al.’s scheme. The informal and formal security analysis of our scheme are given, which demonstrate that our scheme satisfies the desirable security requirements. The presented scheme provides a variety of significant functionalities, in which some features are not considered in the most of existing authentication schemes, such as, user revocation or re-registration and biometric information protection. Compared with several related schemes, our scheme has more secure properties and lower computation cost. It is obviously more appropriate for practical applications in the remote distributed networks.

## Introduction

With the rapid development of Internet, advances in the information and communication technology enhance the quality of online services for distributed networks, which provide the highly useful services to users in a variety of aspects, such as online medicine, online education, online shopping and internet banking [1, 2]. Also there is always interaction between users and servers over a public channel so that design and analysis of secure and efficient authentication scheme have received a considerable attention nowadays [3]. Since the first one was proposed by Lamport, a great number of authentication schemes have been presented, which provide authorized communication between remote entities [4–9]. According to the evidences adopted in the authentication, the existing schemes are divided into two categories: certificate-based and identity-based [10–16]. The former category requires the high computation cost and large storage space for the management of certificate store. Although elliptic curve cryptosystem is introduced, they do not simplify the certificate management so that certificate-based schemes are unacceptable in a real-time application such as multi-media and video conference. To solve the aforementioned problems, Shamir proposed an identity-based public key cryptosystem [17]. But integer factorization problem applied in the Shamir’s scheme is difficult to be implemented efficiently [18]. And then some other identity-based schemes are presented, which are also based on the pairing operation or elliptic curve [19–24]. However, most of them are inefficient because of complicated structures [25–28]. Therefore, secure identity-based authentication schemes that only apply the random numbers and hash function are considered as optimum designs for mobile users and real-time applications.

Furthermore, there are some security vulnerabilities to identity-based authentication schemes in the compromise of passwords and tokens [29–35]. In particular, it is difficult to remember long and random passwords for users, and short passwords are easily broken by simple dictionary attacks because of low entropy. Also it is feasible to extract the information stored in the smart cards by side channel attacks, such as SPA or DPA [36]. To solve these problems, many researchers have combined the biometrics, passwords and tokens to enhance the security of authentication schemes [37–39]. The uniqueness of biometrics in the authentication scheme makes it difficult for adversary to forge the biometric information [40, 41]. And authentication does not request users to remember the biometrics. In fact, biometric characteristics imprinted by users are not exactly the same every time so that directly using them always results in low acceptation of valid users in the biometric-based authentication schemes [42]. Since the failure to authorized users significantly impacts on the availability of schemes, we introduce the fuzzy extractor to reduce the probability of rejection effectively, which is a convenient mechanism to be implemented in the smart card [43, 44].

Meanwhile, conventional authentication schemes are not suitable for the multi-server environment [45, 46]. When single server authenticated schemes are adopted in the multi-server environment, users not only login and access to different remote servers with repetitive registration, but also remember different information about identities and passwords for each server. It decreases the adoption of large network based on the applications. With the assistance of registration center, single registration helps the remote distributed system allow users to access the resources efficiently and conveniently, which is an important consideration in the multi-server architecture. Besides, authentication mechanism is required to achieve a higher level of security in the multi-server environment [47]. There are defects in many multi-server authentication schemes, since users apply the same identities and passwords to login the different servers [48–50]. It gives adversaries opportunities to trace legal users, which usually makes schemes vulnerable to insider attack, masquerade attack and server spoofing attack. For example, Chuang and Chen [51] proposed an anonymous multi-server authenticated key agreement scheme in 2014, and claimed that their scheme not only supported multiple servers but also achieved various security requirements. However, Choi et al. [52] pointed out that Chuang and Chen’s scheme was vulnerable to the smart card attack, user impersonation attack, masquerade attack, DoS attack, and did not achieve the perfect forward secrecy. To achieve the security and efficiency, an authentication scheme for the multi-server environment should meet the following requirements: 1) registration center should be avoided in the authentication phase to avoid the bottlenecks, 2) multiple secret keys in the smart card should not be required to reduce the storage requirement, 3) servers can be easily added on the later stage, and 4) all involved servers may not be trusted [3]. Thus, more work about authenticated key agreement schemes based on the multi-server needs to be studied.

Recently, a user anonymity-preserving biometric-based multi-server authenticated key agreement scheme using smart cards is proposed by Mishra et al. [53], which is applicable for expert systems to achieve the anonymous authentication in multi-server environment. Expert systems have several applications such as security auditing and network management, which emulate or act in all respects with decision-making capabilities of human experts. And Mishra et al. claimed that their scheme satisfied the all security attributes. Unfortunately, according to the cryptanalysis given in this paper, we identify that their scheme does not resist the masquerade attack, replay attack and Denial-of-Service (DoS) attack. We also find that their scheme fails to achieve the perfect forward secrecy. In addition, there is no consideration of the revocation or re-registration phase in the most of existing authentication schemes. To solve these problems, we propose a robust biometric-based multi-server authentication and key agreement scheme. Our scheme improves the Mishra et al.’s scheme and satisfies the desirable security requirements. Also presented scheme provides a variety of significant functionalities, such as anonymity, mutual authentication, session key agreement, perfect forward secrecy, user revocation or re-registration, and biometric information protection. In addition, comparison results show that our scheme has more secure properties, more functionalities and lower computation cost, which make our scheme more appropriate for practical applications in the remote distributed networks.

The remaining of the paper is organized as follows. Next section briefly introduces the threat assumptions, fuzzy extractor and one-way collision-resistant hash function which are adopted in our scheme. Section 3 reviews the Mishra et al.’s scheme. Section 4 mainly discusses the weaknesses of Mishra et al.’s scheme. Section 5 describes the proposed scheme in detail. And then section 6 provides the security, functionality and performance analysis of our algorithm. The last section gives the conclusion.

## Preliminaries

In this section, we describe some concepts about threat assumptions, fuzzy extractor and one-way collision-resistant hash function, which are useful in our scheme.

### Threat assumptions

In this paper, we introduce the Dolev-Yao threat model [54] and consider the risk of side-channel attacks [55] to construct the threat assumptions which are described as follows:

1. Adversary *E* eavesdrops all the communications between user and server over a public channel.
2. Adversary *E* modifies, deletes, resends and reroutes the eavesdropped messages.
3. Adversary *E* may be a malicious user or an outsider in this system.
4. Adversary *E* extracts the sensitive stored information from lost or stolen smart card by examining the power consumption.

### Fuzzy extractor

**Fig 1:**
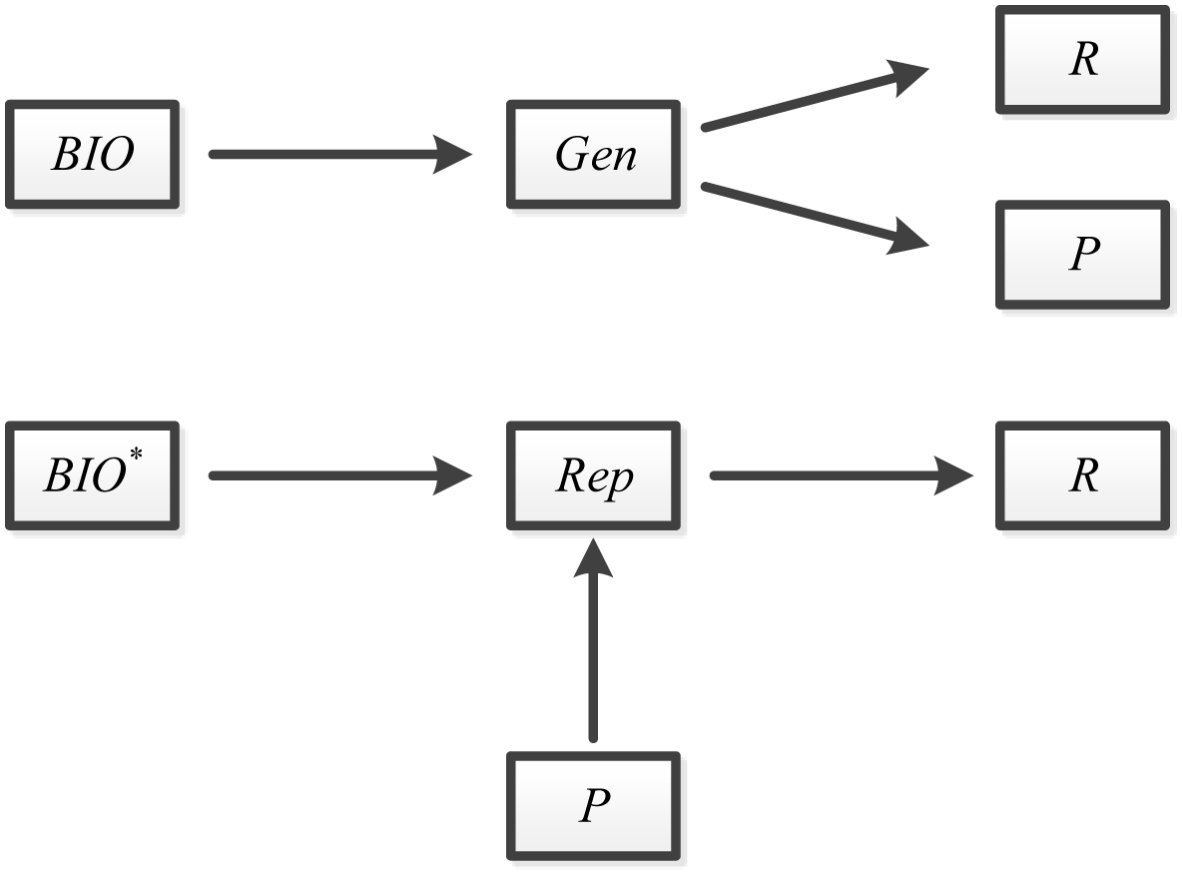
The mechanism of fuzzy extractor.

The mechanism of fuzzy extractor consists of two procedures (*Gen*, *Rep*), which is illustrated in Fig 1.

The function *Gen* is a probabilistic generation procedure, which extracts biometric input *BIO*, and outputs a nearly random binary string *R* ∈ {0, 1}^*l*^ and an auxiliary binary string *P* ∈ {0, 1}*. Also the function *Rep* is a deterministic reproduction procedure allowing to recover *R* with the assistance of corresponding auxiliary string *P* and biometric BIO*. If *dis*(*BIO*, BIO*) ≤ *t* and *Gen*(*BIO*) → 〈*R*, *P*〉, then we have *Rep*(BIO*, *P*) = *R*. Otherwise, there is no guarantee provided by function *Rep*. The error-tolerant makes it dependable to recover nearly uniform randomness *R* with auxiliary string *P* from biometric input BIO*, as long as it remains reasonably close to original input *BIO*. More details about fuzzy extractor are described in the literature [43, 44].

### One-way collision-resistant hash function

The one-way collision-resistant hash function *h* = *h*(*x*) : {0, 1}* → {0, 1}^*n*^ is a deterministic algorithm, which outputs a fixed-length binary string {0, 1}^*n*^ based on the arbitrary length binary string {0, 1}* [56]. It is computationally infeasible to retrieve the input *x* from given hash value and hash function, which is called the one-way property. Also hash function possesses weak/strong collision resistant property. For a given input *x*, finding any input *y* ≠ *x* so that *h*(*x*) = *h*(*y*) is computationally infeasible. For a given pair of inputs (*x*, *y*) with *x* ≠ *y*, then *h*(*x*) = *h*(*y*) is computationally infeasible. The well-known example of hash function is SHA-1. However, Manuel showed that SHA-1 is insecure against the collision attacks in 2011 [57]. So we apply the SHA-2 as secure hash function in our scheme.

## Review of Mishra et al.’s scheme

Recently, Mishra et al. proposed a biometric-based multi-server key agreement scheme using smart cards to achieve the light-weight authentication and user anonymity. There are five phases relating to Mishra et al.’s scheme, which are the server registration phase, user registration phase, login phase, authentication phase and password change phase, respectively. Suppose that *RC* is the trusted third party, which is responsible for the registration of users and servers. Table 1 lists the notations used in their scheme.

**Table 1:** Symbols and notions in Mishra et al.’s scheme.

Symbol	Notion
*U*_*i*_, *S*_*j*_	*i*th user and *j*th server
*RC*, *E*	The registration center and adversary
*ID*_*i*_, *SID*_*j*_	*U*_*i*_’s identity and *S*_*j*_’s identity
*SC*_*i*_, *PW*_*i*_, *BIO*_*i*_	*U*_*i*_’s smart card, password and biometrics
*PSK*, *x*	Pre shared key and master secret key
*h*(⋅), *H*(⋅)	Hash function and biohash function
⊕, ||	XOR operation and concatenation operation

### Server registration phase

1. The server *S*_*j*_ sends a join message to the *RC*.
2. After receiving the join message, *RC* replies with the pre shared key (*PSK*) to the server *S*_*j*_ through a secure channel.
3. Upon receiving the *PSK*, the authorized server *S*_*j*_ uses this key to authorize the legitimate users.

### User registration phase

1. The new user *U*_*i*_ selects the identity *ID*_*i*_ and password *PW*_*i*_. Then *U*_*i*_ generates a random number *N*_*i*_, computes *W*_1_ = *h*(*PW*_*i*_||*N*_*i*_) and *W*_2_ = *h*(*ID*_*i*_ ⊕ *N*_*i*_), and sends the registration request message {*ID*_*i*_, *W*_1_, *W*_2_} to the *RC* via a secure channel.
2. After receiving the registration request message, *RC* computes *A*_*i*_ = *h*(*ID*_*i*_||*x*|*T*_*r*_|), *B*_*i*_ = *h*(*A*_*i*_), *X*_*i*_ = *B*_*i*_ ⊕ *W*_1_, *Y*_*i*_ = *h*(*PSK*) ⊕ *W*_2_ and *Z*_*i*_ = *PSK* ⊕ *A*_*i*_, where *T*_*r*_ is the registration time. And *RC* issues the smart card *SC*_*i*_ to the user *U*_*i*_, which contains {*X*_*i*_, *Y*_*i*_, *Z*_*i*_, *h*(⋅)} via a secure channel.
3. Upon receiving the *SC*_*i*_, *U*_*i*_ imprints the personal biometrics *BIO*_*i*_ at the sensor, and computes *N* = *N*_*i*_ ⊕ *H*(*BIO*_*i*_), *V* = *h*(*ID*_*i*_||*N*_*i*_||*PW*_*i*_). Finally, the user *U*_*i*_ stores {*X*_*i*_, *Y*_*i*_, *Z*_*i*_, *N*, *V*, *h*(⋅)} into the *SC*_*i*_.

### Login phase

1. *U*_*i*_ inserts the *SC*_*i*_ into the smart card reader and inputs the identity *ID*_*i*_ and password *PW*_*i*_, and imprints the biometrics *BIO*_*i*_ at the sensor.
2. *SC*_*i*_ computes *N*_*i*_ = *N* ⊕ *H*(*BIO*_*i*_) and checks whether *h*(*ID*_*i*_||*N*_*i*_||*PW*_*i*_) = *V* holds. If it holds, *SC*_*i*_ further compute *W*_1_ = *h*(*PW*_*i*_||*N*_*i*_), *W*_2_ = *h*(*ID*_*i*_ ⊕ *N*_*i*_), *B*_*i*_ = *X*_*i*_ ⊕ *W*_1_ and *h*(*PSK*) = *Y*_*i*_ ⊕ *W*_2_.
3. *SC*_*i*_ generates a random number *n*_1_, and computes *M*_1_ = *h*(*PSK*) ⊕ *n*_1_, *M*_2_ = *ID*_*i*_ ⊕ *h*(*n*_1_||*B*_*i*_) and *M*_3_ = *h*(*ID*_*i*_||*n*_1_||*B*_*i*_).
4. *U*_*i*_ sends the login request message {*Z*_*i*_, *M*_1_, *M*_2_, *M*_3_} to *S*_*j*_ over a public channel.

### Authentication phase

1. When receiving the login request message from *SC*_*i*_, *S*_*j*_ immediately computes *A*_*i*_ = *Z*_*i*_ ⊕ *PSK*, *n*_1_ = *M*_1_ ⊕ *h*(*PSK*), *ID*_*i*_ = *M*_2_ ⊕ *h*(*n*_1_||*h*(*A*_*i*_)), and verifies whether *h*(*ID*_*i*_||*n*_1_||*B*_*i*_) is consistent with *M*_3_. If this verification holds, *S*_*j*_ generates a random number *n*_2_ and computes the session secret key *SK*_*ji*_ = *h*(*ID*_*i*_||*SID*_*j*_||*B*_*i*_||*n*_1_||*n*_2_), *M*_4_ = *n*_2_ ⊕ *h*(*ID*_*i*_||*n*_1_), *M*_5_ = *h*(*SK*_*ji*_||*n*_1_||*n*_2_). Then *S*_*j*_ sends the authentication request message {*SID*_*j*_, *M*_4_, *M*_5_} to *SC*_*i*_ via a public channel.
2. Upon receiving the authentication request message, *SC*_*i*_ retrieves *n*_2_ = *M*_4_ ⊕ *h*(*ID*_*i*_||*N*_1_), *SK*_*ij*_ = *h*(*ID*_*i*_||*SID*_*j*_||*B*_*i*_||*n*_1_||*n*_2_) and then checks whether *h*(*SK*_*ij*_||*n*_1_||*n*_2_) = *M*_5_ holds. If it holds, *SC*_*i*_ computes *M*_6_ = *h*(*SK*_*ij*_||*n*_2_||*n*_1_) and delivers the authentication reply {*M*_6_} to *S*_*j*_ via a public channel.
3. *S*_*j*_ verifies whether *h*(*SK*_*ij*_||*n*_2_||*n*_1_) = *M*_6_ holds. If this verification holds, *S*_*j*_ can now use the session key *SK*_*ij*_ to communicate with *U*_*i*_.

### Password change phase

1. *U*_*i*_ inputs the *ID*_*i*_, *PW*_*i*_ and imprints his biometrics *BIO*_*i*_ at the sensor. *SC*_*i*_ computes *N*_*i*_ = *N* ⊕ *h*(*BIO*_*i*_) and checks whether *h*(*ID*_*i*_||*N*_*i*_||*PW*_*i*_) = *V* holds.
2. If the verification holds, *U*_*i*_ choose the new password $PW_{i}^{new}$. *SC*_*i*_ computes *W*_1_ = *h*(*PW*_*i*_||*N*_*i*_), $W_{1}^{new}=h(PW_{i}^{new}\left|\right|N_{i})$, $X_{i}^{new}=X_{i}⊕W_{1}⊕W_{1}^{new}$ and $V_{i}^{new}=h(ID_{i}\left|\right|N_{i}||PW_{i}^{new})$.
3. *SC*_*i*_ replaces *X*_*i*_ with $X_{i}^{new}$ and *V*_*i*_ with $V_{i}^{new}$ in the memory.

## Cryptanalysis of Mishra et al.’s scheme

This section presents a cryptanalysis of Mishra et al.’s scheme and demonstrates that their scheme is still vulnerable to the masquerade attack, replay attack and Denial-of-Service attack. Also their scheme fails to achieve the perfect forward secrecy. Furthermore, Mishra et al.’s scheme does not provide the functionality of revocation/re-registration for user’s requirements.

### Masquerade attack

**Fig 2:**
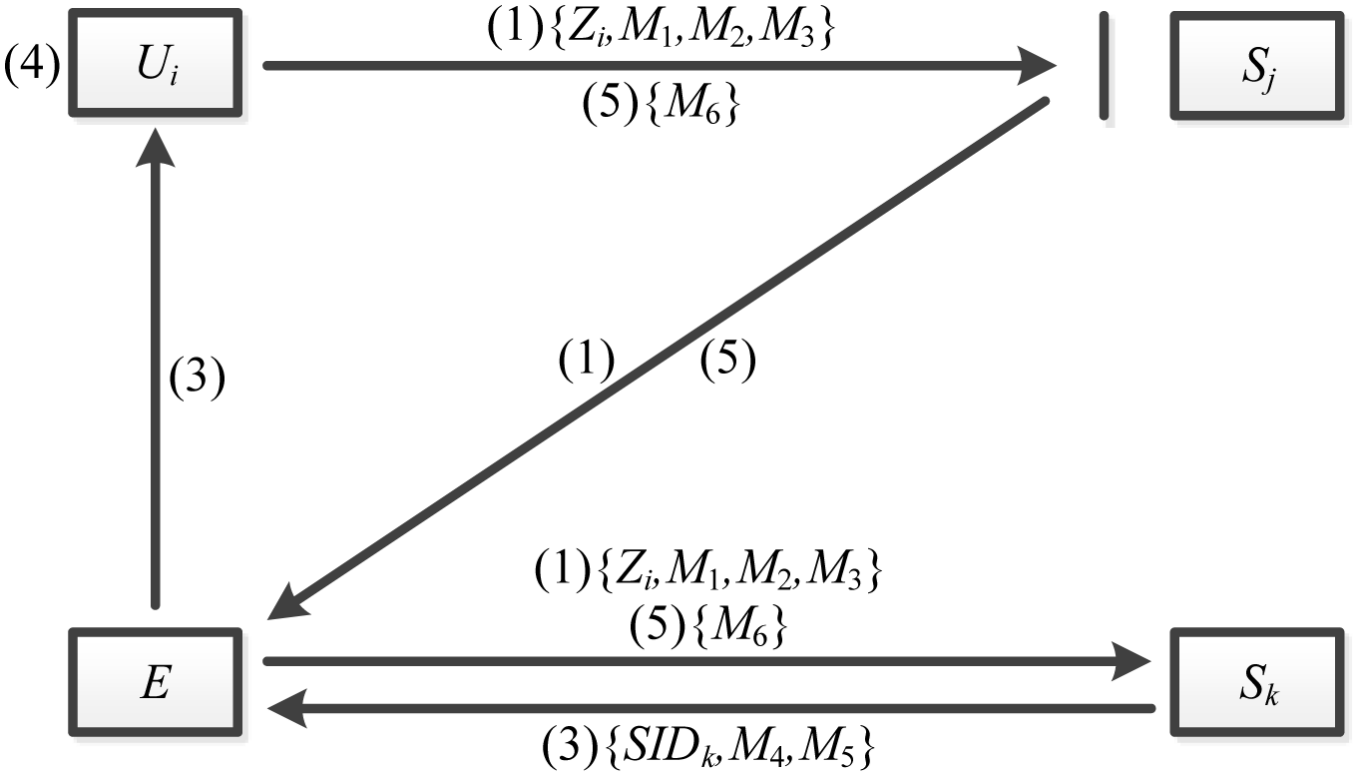
The masquerade attack on Mishra et al.’s scheme.

Mishra et al.’s scheme is vulnerable to the masquerade attack. More narrowly, adversary *E* can be authenticated by another server *S*_*k*_ using the messages that user *U*_*i*_ sends to the server *S*_*j*_ for the authentication. Fig 2 shows the masquerade attack on Mishra et al.’s scheme.

First, *U*_*i*_ inserts the smart card and sends a login request message (1) to the *S*_*j*_ when he wants to be authenticated by *S*_*j*_. After intercepting the login request message, *E* sends it to another server *S*_*k*_. The message (1) does not include the information about the *S*_*j*_ as follows.

$$\begin{array}{c} Message\left(1\right)=\left\{Z_{i},M_{1},M_{2},M_{3}\right\}, \end{array}$$

where *Z*_*i*_ = *PSK* ⊕ *h*(*ID*_*i*_||*x*||*T*_*r*_), *M*_1_ = *h*(*PSK*) ⊕ *n*_1_, *M*_2_ = *ID*_*i*_ ⊕ *h*(*n*_1_||*B*_*i*_) and *M*_3_ = *h*(*ID*_*i*_||*n*_1_||*B*_*i*_). Therefore *S*_*k*_ executes the operation (2) and sends the authentication request message (3) to the *E* without any suspicion of the attack.

Then *E* transmits the message (3) to the *U*_*i*_. And *U*_*i*_ does not check the identity of the server. He only checks the sameness with the *SID*_*k*_ in the *M*_5_ and the *SID*_*k*_ in the message (3) as follows.

$$\begin{array}{c} Message\left(3\right)=\left\{SID_{k},M_{4},M_{5}\right\}, \end{array}$$

where *M*_4_ = *n*_2_ ⊕ *h*(*ID*_*i*_||*n*_1_), *M*_5_ = *h*(*SK*_*ki*_||*n*_1_||*n*_2_) and *SK*_*ki*_ = *h*(*ID*_*i*_||*SID*_*k*_||*B*_*i*_||*n*_1_||*n*_2_). So *U*_*i*_ also executes the operation (4) and sends the authentication reply message (5) to the *S*_*j*_ without any suspicion of the attack.

Finally, *E* intercepts the message (5) and transmits it to the *S*_*k*_. Therefore *E* can be authenticated by *S*_*k*_. In conclusion, adversary *E* can masquerade as a legitimate user to log in to the server *S*_*k*_ so that Mishra et al.’s scheme becomes vulnerable to the masquerade attack.

In their scheme, *S*_*k*_ cannot check whether *U*_*i*_ wants to be authenticated by *S*_*k*_. Thus *S*_*k*_ authenticates all legitimate messages though these message are not sent to *S*_*k*_. Similarly, *U*_*i*_ does not check whether *S*_*k*_ wants to be authenticated with *U*_*i*_. He only checks whether *SID* in the message (3) and *SID* in the *M*_5_ are the same.

To meet these challenges, the destination of message needs to be added to the login request message (1) and the authentication request message (3). So we add the information about *SID*_*j*_ of server *S*_*j*_ to the message (1), which means that *U*_*i*_ want to be authenticated by *S*_*j*_, not *S*_*k*_. Meanwhile, the information about *AID*_*i*_ of user *U*_*i*_ needs to be added to the message (3), which means that *S*_*j*_ wants to be authenticated by anonymous *U*_*i*_.

### Replay attack

In the same way, Mishra et al.’s scheme is vulnerable to the replay attack. In particular, adversary *E* logs into the server *S*_*j*_ with previous login request message (1). Upon receiving previous message (1), *S*_*j*_ calculates *A*_*i*_ = *Z*_*i*_ ⊕ *PSK*, *n*_1_ = *M*_*P*1_ ⊕ *h*(*PSK*), *ID*_*i*_ = *M*_*P*2_ ⊕ *h*(*n*_1_||*h*(*A*_*i*_)), and verifies whether *h*(*ID*_*i*_||*n*_1_||*B*_*i*_) = *M*_*P*3_ holds without any suspicion of the attack. Since the verification holds, *S*_*j*_ authenticates *E* and *E* logs into the server *S*_*j*_. Thus Mishra et al.’s scheme becomes vulnerable to the replay attack.

In their scheme, *S*_*j*_ does not check the freshness of login request message. So *S*_*j*_ authenticates all legitimate login request messages though these messages are not fresh.

As a practical solution to prevent the replay attack, adding the timestamp to the message (1) helps server *S*_*j*_ verify the freshness of login request message.

### Denial-of-Service attack

**Fig 3:**
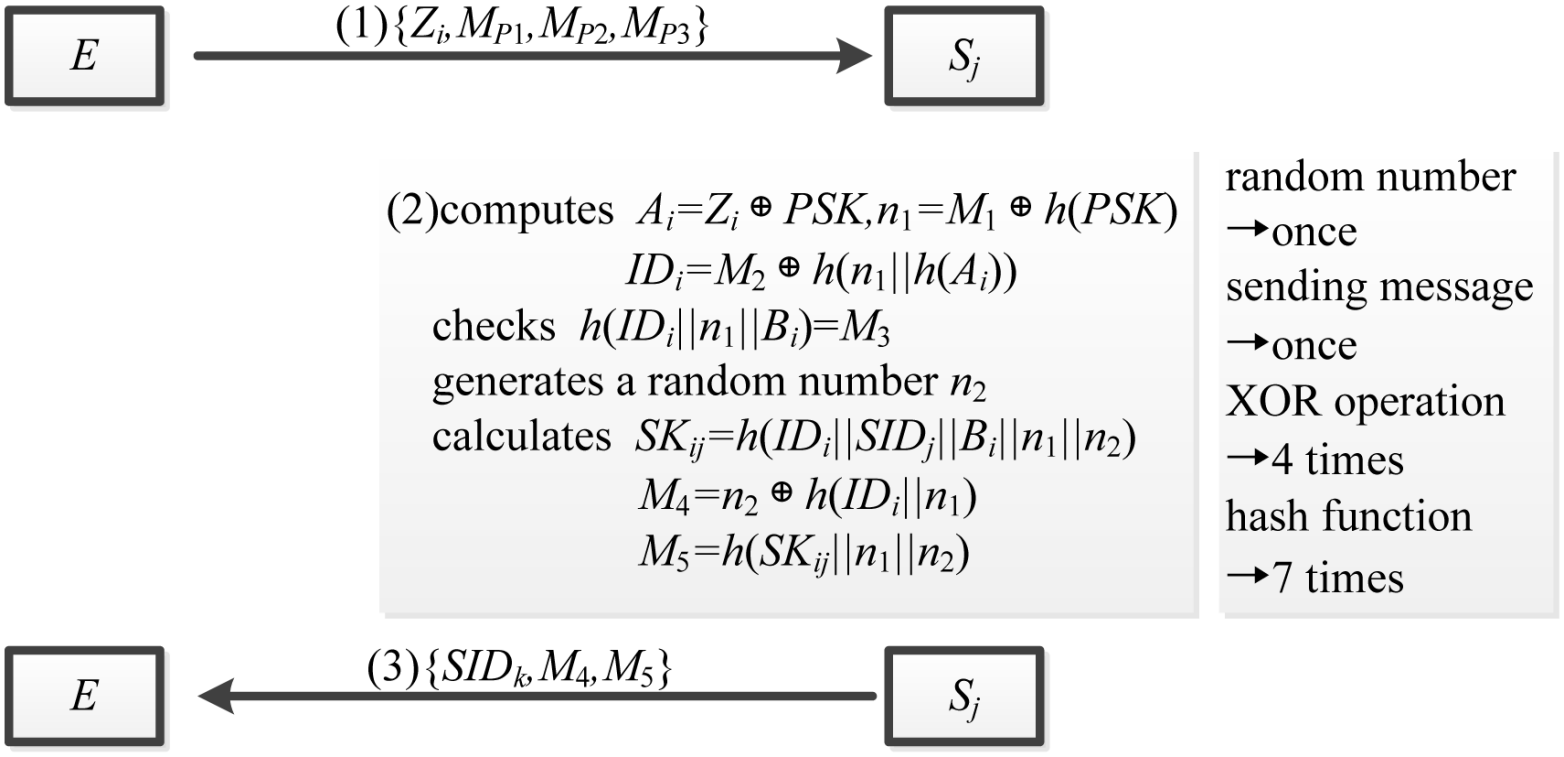
The DoS attack on Mishra et al.’s scheme.

Although the means and targets may vary, DoS attack is generally an attempt to make network resource or machines unavailable for intended users, which temporarily or indefinitely interrupts or suspends the services of a host connected to the networks. In the Mishra et al.’s scheme, an adversary *E* can carry out the DoS attack without difficulty. Fig 3 describes the procedure and effect of the DoS attack on Mishra et al.’s scheme.

In particular, *E* collects the previous login request message {*Z*_*i*_, *M*_*P*1_, *M*_*P*2_, *M*_*P*3_} from the user *U*_*i*_ and then forwards it to the server *S*_*j*_. Upon receiving the login request, *S*_*j*_, as always, executes the operation (2) which includes producing the random number once, sending message once, calculating the XOR operation 4 times, and performing the hash function 7 times. By applying the intercepted login request messages repeatedly, adversary *E* can make the services of network resource or servers unavailable. Therefore Mishra et al.’s scheme becomes vulnerable to the DoS attack.

The reason for this result is that server *S*_*j*_ cannot check the freshness of login request message from the user *U*_*i*_. *S*_*j*_ does not know whether the received messages are outdated so that it executes the operation (2) once receiving the login request message.

To resist the DoS attack, the timestamp needs to be added to the login request message. So we add the timestamp to the message (1), which helps the servers check the freshness of messages.

### No perfect forward secrecy

The perfect forward secrecy means that if one of long-term keys is compromised, a session key which is derived from these long-term keys will not be compromised in the future [58]. Unfortunately, Mishra et al.’s scheme does not achieve the perfect forward secrecy. So adversary *E* can calculate all session keys between the user *U*_*i*_ and server *S*_*j*_ if he knows one of long-term keys, such as *A*_*i*_.

First, *E* intercepts the *Z*_*i*_, *SID*_*j*_, *M*_*P*1_, *M*_*P*2_and *M*_*P*4_ from message (1) and message (3) in the previous communication between *U*_*i*_ and *S*_*j*_. Next, adversary knows one of long-term keys *A*_*i*_ so that he can compute *PSK* from *PSK* = *A*_*i*_ ⊕ *Z*_*i*_ and *B*_*i*_ from *B*_*i*_ = *h*(*A*_*i*_). Then, *E* further calculate *n*_*P*1_ from *n*_*P*1_ = *M*_*P*1_ ⊕ *h*(*PSK*), *ID*_*i*_ from *ID*_*i*_ = *M*_*P*2_ ⊕ *h*(*n*_*P*1_||*B*_*i*_), and *n*_*P*2_ from *n*_*P*2_ = *M*_*P*4_ ⊕ *h*(*ID*_*i*_||*N*_*P*1_). Finally, adversary *E* acquires the all previous session keys from *SK*_*Pji*_ = *h*(*ID*_*i*_||*SID*_*j*_||*B*_*i*_||*n*_1_||*n*_2_). Therefore Mishra et al.’s scheme does not achieve the perfect forward secrecy.

In their scheme, *A*_*i*_ is a shared key between *RC* and *U*_*i*_, which is calculated from *A*_*i*_ = *h*(*ID*_*i*_||*x*||*T*_*r*_). *RC* stores the information about *A*_*i*_ and *h*(*A*_*i*_) in the smart card *SC*_*i*_. The value of *A*_*i*_ is invariable even if *U*_*i*_ updates the password. So *A*_*i*_ is treated as one of long-term keys. From the above, it is demonstrated that there are some defects during the generation of session keys.

To solve this problem, we need to add another secret information, such as *PSK*, to the generation of session keys. Also it is necessary to prevent adversary *E* from calculating all session keys by using long-term key *A*_*i*_ and information in the public channel.

### No user revocation/re-registration phase

There is no user revocation/re-registration phase in the Mishra et al.’s scheme so that user *U*_*i*_ cannot revoke his privilege or re-register when his smart card *SC*_*i*_ is stolen or lost. To promote the functionality of scheme, we design the corresponding revocation/re-registration phase for the user’s requirements. And more details are showed in the Section 5.6.

## The proposed scheme

Based on the cryptanalysis of Mishra et al.’s scheme, we present a novel robust biometric-based multi-server authentication and key agreement scheme which consists of six phases: server registration phase, user registration phase, login phase, authentication phase, password change phase and revocation/re-registration phase. There are also three participants, user *U*_*i*_, server *S*_*j*_ and registration center *RC*. Table 2 lists the notations applied in our scheme.

**Table 2:** Symbols and notions in our scheme.

Symbol	Notion
*U*_*i*_, *S*_*j*_	*i*th user and *j*th server
*RC*, *E*	The registration center and adversary
*ID*_*i*_, *AID*_*i*_, *SID*_*j*_	*U*_*i*_’s identity, dynamic identity and *S*_*j*_’s identity
*SC*_*i*_, *PW*_*i*_, *BIO*_*i*_	*U*_*i*_’s smart card, password and biometrics
*R*_*i*_, *P*_*i*_	*U*_*i*_’s nearly random binary string and auxiliary binary string
*PSK*, *x*	Pre shared key and master secret key
*h*(⋅), ⊕, ||	Hash function, XOR operation and concatenation operation

The proposed scheme improves the Mishra et al.’s scheme in the several aspects: 1) it resists the masquerade attack by adding the destination of messages, 2) it appends the timestamp to prevent the Denial-of-Service (DoS) attack, 3) it introduces pre shared key (*PSK*) into generation of session keys to achieve the perfect forward secrecy, 4) it provides the revocation/re-registration phase for user’s requirements, and 5) it enhances the performance of scheme, especially login phase. The details are described in the following subsections.

### Server registration phase

**Fig 4:**
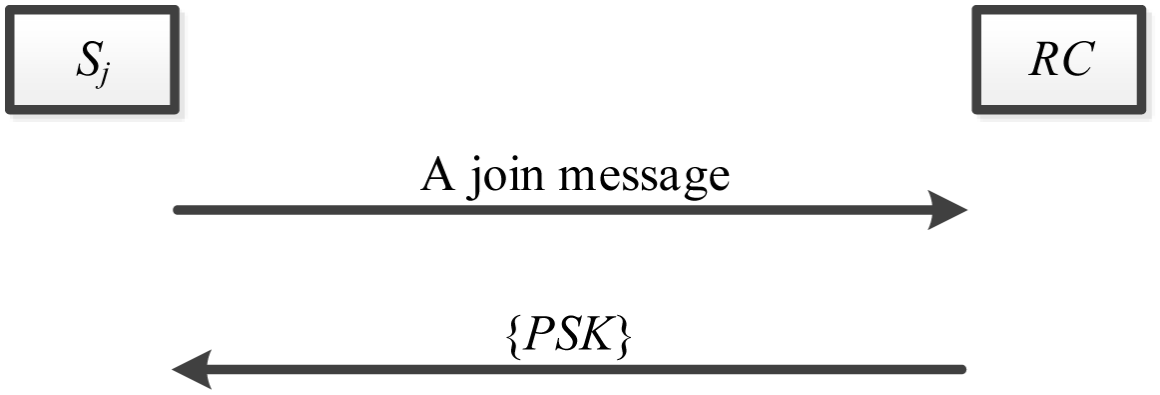
The server registration phase.

The server registration phase is illustrated in Fig 4 and explained as follows.

1. The server *S*_*j*_ sends a join request message to the registration center *RC*, if it wants to become an authorized server in the system.
2. After receiving the join request message, *RC* authorizes the server and replies with the pre shared key (*PSK*) to the server *S*_*j*_ by applying the Key Exchange Protocol (IKEv2) through a secure channel.
3. Upon receiving the secret key *PSK*, authorized server *S*_*j*_ uses the shared information, such as *PSK* and *h*(*PSK*), to check the user’s legitimacy in the authentication phase.

### User registration phase

**Fig 5:**
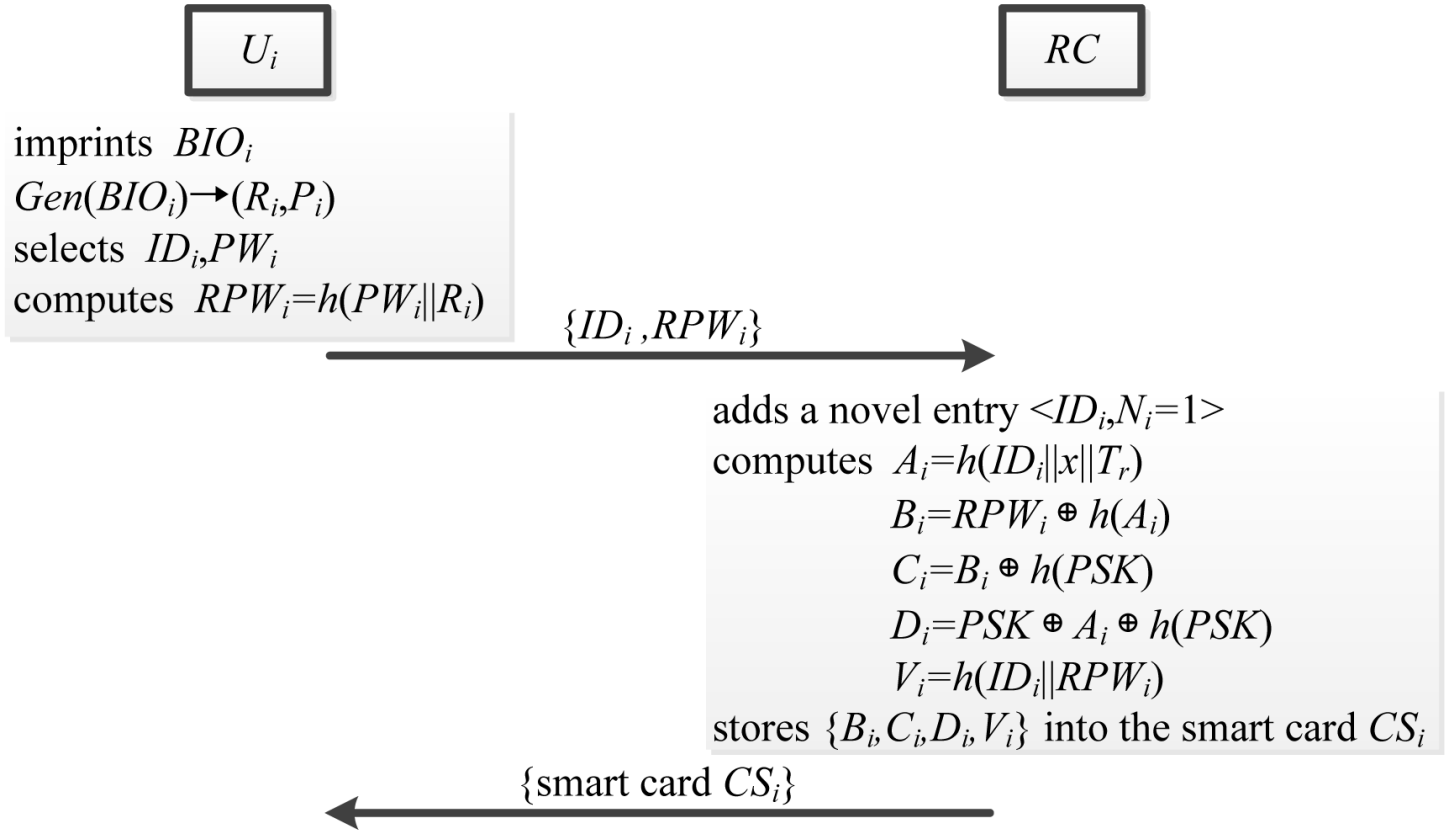
The user registration phase.

The new user *U*_*i*_ needs to execute the user registration phase with the registration center *RC* via a secure channel. The user registration phase is showed in Fig 5 and described as follows.

1. First, *U*_*i*_ imprints the personal biometric information *BIO*_*i*_ at the sensor. After that, sensor sketches *BIO*_*i*_, extracts (*R*_*i*_, *P*_*i*_) from *Gen*(*BIO*_*i*_) → (*R*_*i*_, *P*_*i*_), and stores *P*_*i*_ in the memory. Next, *U*_*i*_ selects the identity *ID*_*i*_ and password *PW*_*i*_, and computes *RPW*_*i*_ = *h*(*PW*_*i*_||*R*_*i*_). Finally, *U*_*i*_ sends the registration request message {*ID*_*i*_, *RPW*_*i*_} to the *RC* via a secure channel.
2. After receiving the registration request message, *RC* adds a novel entry 〈*ID*_*i*_, *N*_*i*_ = 1〉 to the database, where *N*_*i*_ means the times of user registration. And then *RC* computes *A*_*i*_ = *h*(*ID*_*i*_||*x*||*T*_*r*_), *B*_*i*_ = *RPW*_*i*_ ⊕ *h*(*A*_*i*_), *C*_*i*_ = *B*_*i*_ ⊕ *h*(*PSK*), *D*_*i*_ = *PSK* ⊕ *A*_*i*_ ⊕ *h*(*PSK*) and *V*_*i*_ = *h*(*ID*_*i*_||*RPW*_*i*_), where *T*_*r*_ is the registration time.
3. *RC* issues the smart card *SC*_*i*_ to the user *U*_*i*_, which contains {*B*_*i*_, *C*_*i*_, *D*_*i*_, *V*_*i*_} over a secure channel.
4. Upon receiving the *SC*_*i*_, *U*_*i*_ stores *P*_*i*_ into the *SC*_*i*_ and initializes the authentication environments.

### Login phase

**Fig 6:**
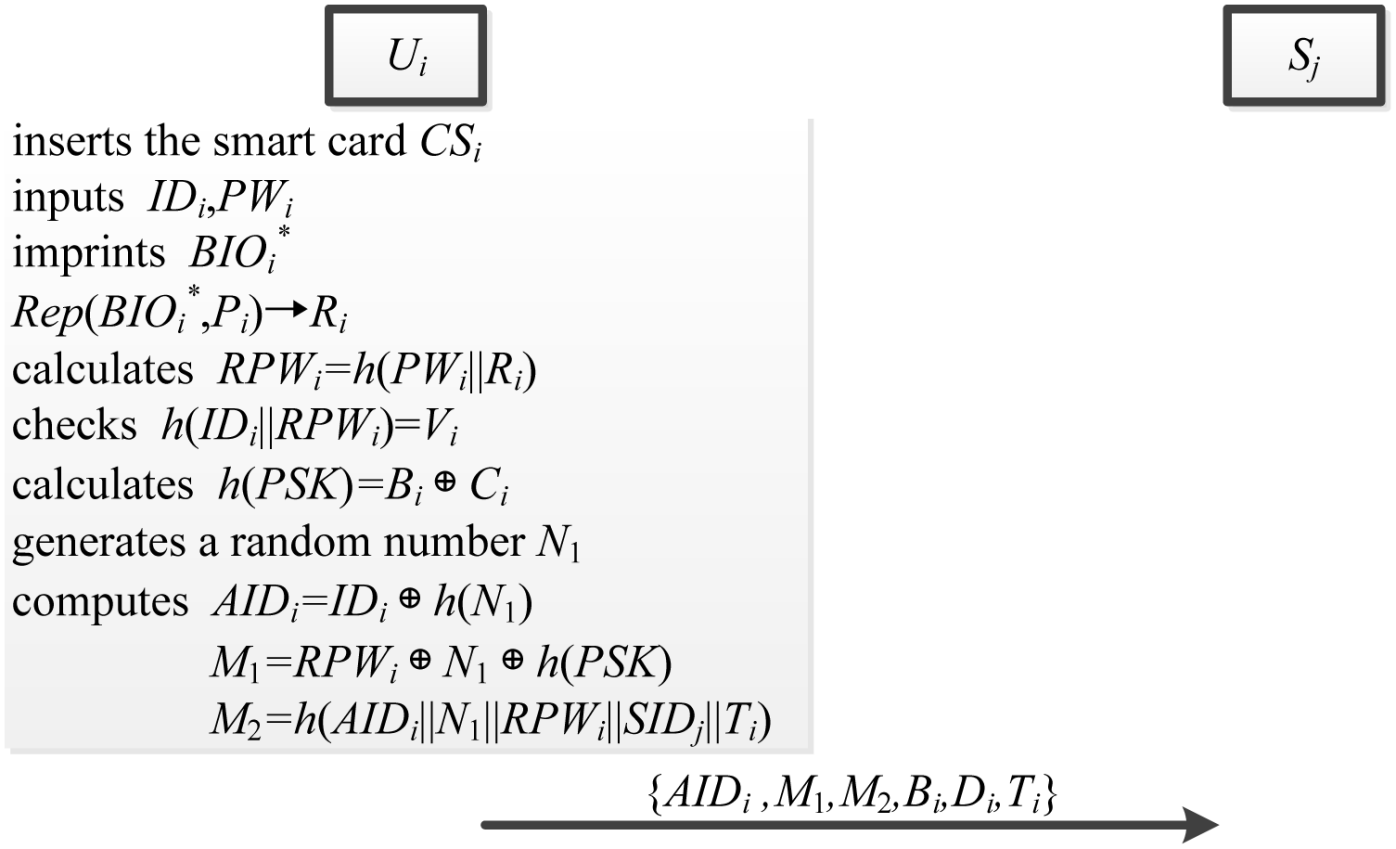
The login phase.

During the login phase, smart card *SC*_*i*_ can check an error event immediately by using the identification, password, and biometric information. The login phase is illustrated in Fig 6 and explained as follows.

1. *U*_*i*_ inserts the *SC*_*i*_ into the smart card reader, inputs the identity *ID*_*i*_ and password *PW*_*i*_, and imprints the biometrics $BIO_{i}^{*}$ at the sensor. After that, sensor sketches $BIO_{i}^{*}$ and recovers *R*_*i*_ from $Rep\left(BIO_{i}^{*},P_{i}\right)\to R_{i}$.
2. *SC*_*i*_ calculates *RPW*_*i*_ = *h*(*PW*_*i*_||*R*_*i*_) and checks whether *h*(*ID*_*i*_||*RPW*_*i*_) = *V*_*i*_ holds. If it holds, *SC*_*i*_ further calculates *h*(*PSK*) = *B*_*i*_ ⊕ *C*_*i*_.
3. *SC*_*i*_ generates a random number *N*_1_, and computes *AID*_*i*_ = *ID*_*i*_ ⊕ *h*(*N*_1_), *M*_1_ = *RPW*_*i*_ ⊕ *N*_1_ ⊕ *h*(*PSK*) and *M*_2_ = *h*(*AID*_*i*_||*N*_1_||*RPW*_*i*_||*SID*_*j*_||*T*_*i*_), where *T*_*i*_ is additional timestamp.
4. *SC*_*i*_ sends the login request message {*AID*_*i*_, *M*_1_, *M*_2_, *B*_*i*_, *D*_*i*_, *T*_*i*_} to *S*_*j*_ via a public channel.

### Authentication phase

**Fig 7:**
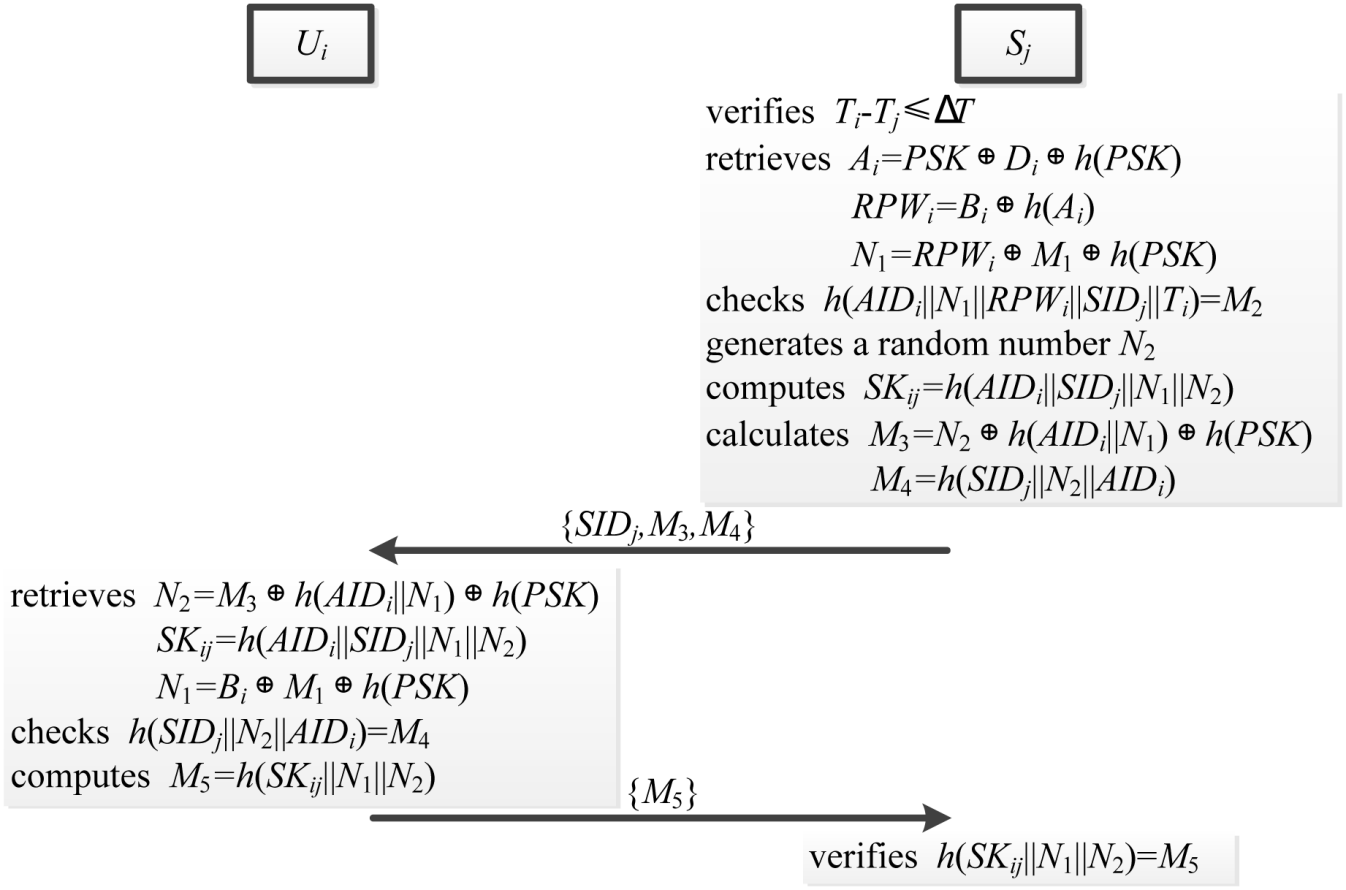
The authentication phase.

In the authentication phase, server *S*_*j*_ confirms the destination and freshness of login request message. The authentication phase is showed in Fig 7 and described as follows.

1. When receiving the login request message from *U*_*i*_, server *S*_*j*_ verifies whether *T*_*i*_ − *T*_*j*_ ≤ *ΔT* is valid, where *ΔT* is the time interval and *T*_*j*_ is the time when *S*_*j*_ receives the login request message. If it holds, *S*_*j*_ continues to perform the next step. Otherwise, the login request will be rejected by *S*_*j*_.
2. *S*_*j*_ retrieves *A*_*i*_ = *D*_*i*_ ⊕ *PSK* ⊕ *h*(*PSK*), *RPW*_*i*_ = *B*_*i*_ ⊕ *h*(*A*_*i*_), *N*_1_ = *RPW*_*i*_ ⊕ *M*_1_ ⊕ *h*(*PSK*), and verifies whether *h*(*AID*_*i*_||*N*_1_||*RPW*_*i*_||*SID*_*j*_||*T*_*i*_) is consistent with *M*_2_.
3. If this verification holds, *S*_*j*_ generates a random number *N*_2_, and computes the session secret key *SK*_*ij*_ = *h*(*AID*_*i*_||*SID*_*j*_||*N*_1_||*N*_2_).
4. *S*_*j*_ calculates *M*_3_ = *N*_2_ ⊕ *h*(*AID*_*i*_||*N*_1_) ⊕ *h*(*PSK*) and *M*_4_ = *h*(*SID*_*j*_||*N*_2_||*AID*_*i*_), and sends the authentication request message {*SID*_*j*_, *M*_3_, *M*_4_} to *U*_*i*_ via a public channel.
5. Upon receiving the authentication request, *SC*_*i*_ retrieves *N*_2_ = *M*_3_ ⊕ *h*(*AID*_*i*_||*N*_1_) ⊕ *h*(*PSK*), *SK*_*ij*_ = *h*(*AID*_*i*_||*SID*_*j*_||*N*_1_||*N*_2_) and then checks whether *h*(*SID*_*j*_||*N*_2_||*AID*_*i*_) = *M*_4_ holds. If it holds, *SC*_*i*_ computes *M*_5_ = *h*(*SK*_*ij*_||*N*_1_||*N*_2_) and delivers the authentication reply {*M*_5_} to *S*_*j*_ via a public channel.
6. *S*_*j*_ verifies whether *h*(*SK*_*ij*_||*N*_1_||*N*_2_) = *M*_5_ holds. If this verification holds, *S*_*j*_ uses the session key *SK*_*ij*_ to communicate with *U*_*i*_. Otherwise, authentication will be rejected by *S*_*j*_.

### Password change phase

During the password change phase, *U*_*i*_ updates the password without any assistance from server *S*_*j*_ and registration center *RC*. This phase consists of the following steps.

1. *U*_*i*_ inputs *ID*_*i*_ and *PW*_*i*_, and imprints his biometrics $BIO_{i}^{*}$ at sensor. After that, the sensor sketches $BIO_{i}^{*}$ and recovers *R*_*i*_ from $Rep\left(BIO_{i}^{*},P_{i}\right)\to R_{i}$.
2. *SC*_*i*_ calculates *RPW*_*i*_ = *h*(*PW*_*i*_||*R*_*i*_) and checks whether *h*(*ID*_*i*_||*RPW*_*i*_) = *V*_*i*_ holds. If the verification holds, *SC*_*i*_ asks *U*_*i*_ for a new password. Otherwise, password change phase is terminated immediately by *SC*_*i*_.
3. *U*_*i*_ inputs new password $PW_{i}^{new}$ and *SC*_*i*_ further computes $RPW_{i}^{new}=h(PW_{i}^{new}\left|\right|R_{i})$, $B_{i}^{new}=B_{i}⊕RPW_{i}⊕RPW_{i}^{new}$, $C_{i}^{new}=C_{i}⊕RPW_{i}⊕RPW_{i}^{new}$ and $V_{i}^{new}=h(ID_{i}||RPW_{i}^{new})$.
4. *SC*_*i*_ replaces *B*_*i*_ with $B_{i}^{new}$, *C*_*i*_ with $C_{i}^{new}$ and *V*_*i*_ with $V_{i}^{new}$ in the memory.

### User revocation/re-registration phase

The functionality of user revocation/re-registration helps user *U*_*i*_ revoke his privilege or re-register when his smart card *SC*_*i*_ is stolen or lost. If *U*_*i*_ wants to revoke his privilege, he needs to send a revocation request message, his smart card and verification message {*RPW*_*i*_} to the registration center *RC* over a secure channel. *RC* verifies whether *U*_*i*_ is valid. If it holds, *RC* further modifies the corresponding entry by setting 〈*ID*_*i*_, *N*_*i*_ = 0〉. Similarly, upon receiving a re-registration request message via a secure channel, *RC* executes the steps described in the section 5.2 and replaces 〈*ID*_*i*_, *N*_*i*_ = *N*_*i*_ + 1〉 with 〈*ID*_*i*_, *N*_*i*_〉 to help *U*_*i*_ re-register. The user revocation or re-registration phase makes our scheme more robust than other related schemes in the functionality.

## Analysis of our scheme

An authentication and key agreement scheme has three important requirements: security, functionality and performance. It is necessary to analyze the proposed scheme from three aspects mentioned above. In this section, we explain how the proposed scheme is satisfied with these requirements, and compare our scheme with other related multi-server authentication and key agreement schemes.

### Informal security analysis

In this section, we assume that adversary *E* has the capacity which is assumed in Section 2.1. Also we analyze the strength of the proposed scheme against the following common attacks through informal security analysis.

#### Resistance to replay attack

The replay attack means that adversary *E* intercepts the transmitted messages for making use of these data in some manner, which involves copying and possibly altering the data in various ways. Although adversary *E* intercepts the previous login request message {*AID*_*i*_, *M*_1_, *M*_2_, *B*_*i*_, *D*_*i*_, *T*_*i*_} and sends it to server *S*_*j*_ repeatedly, *S*_*j*_ verifies the legality of message by checking *T*_*i*_ and *N*_1_ as follows.

$$\begin{array}{c} M_{2}=h(AID_{i}\left|\right|N_{1}||RPW_{i}||SID_{j}\left|\right|T_{i}), \end{array}$$

where *T*_*i*_ and *N*_1_ are different in every session so that *E* is not authenticated by *S*_*j*_. So our scheme is secure against the replay attack by adding the timestamp *T*_*i*_ and random nonce *N*_1_.

#### Resistance to modification attack

Though adversary *E* intercepts the transmitted messages and attempts to modify them for authentication, proposed scheme verifies whether received messages are modified with the help of one-way hash function. And *E* cannot retrieve *N*_1_, *N*_2_ and *PSK* from intercepted messages so that he does not have the capabilities to generate a legitimate authentication message. Therefore, our scheme prevents the modification attack.

#### Resistance to stolen-verifier attack

In the proposed scheme, Registration center *RC* and servers do not possess the user’s password or biometrics so that adversary *E* cannot steal the password-verifier or biometrics-verifier about legitimate users even if he has the authority to access the database of the *RC* and servers. Thus, our scheme resists the stolen-verifier attack.

#### Resistance to off-line guessing attack

With the assistance of the side-channel attacks such as SPA or DPA, adversary *E* obtains *B*_*i*_, *C*_*i*_, *D*_*i*_ and *V*_*i*_. But he cannot verify the user’s password in the off-line environment without *BIO*_*i*_, *PSK*, *x* and *N*_1_. Also user’s password is protected by one-way hash function, such as, *h*(*PW*_*i*_||*R*_*i*_), where *R*_*i*_ possesses high entropy. Moreover, there is no the same biometric templates between any two people. In conclusion, our scheme is secure against the off-line guessing attack.

#### Resistance to forgery attack

The forgery attack means that legitimate yet malicious user *E* attempts to forge another legitimate user for login and authentication. In the communication between server *S*_*j*_ and user *U*_*i*_, *U*_*i*_’s real identity *ID*_*i*_ is protected by anonymous identity *AID*_*i*_, such as *AID*_*i*_ = *ID*_*i*_ ⊕ *h*(*N*_1_). Furthermore, random nonce *N*_1_ changes in every session. So malicious user *E* cannot acquire another legitimate user’s real identity *ID*_*i*_. As a result, our scheme prevents the forgery attack.

#### Resistance to insider attack

Malicious insider *E* is familiar with system policies or procedures, and has an authorized system access, who tries to obtain user’s private information such as password and biometrics. *RC* cannot retrieve the password *PW*_*i*_ or biometrics *BIO*_*i*_ from *RPW*_*i*_ = *h*(*PW*_*i*_||*R*_*i*_). Moreover *RC* does not store *RPW*_*i*_ in the database. Thus, our scheme resists the insider attack.

#### Resistance to masquerade attack

Under this attack, adversary *E* is authenticated by server *S*_*j*_ with a fake or real identity. In Mishra et al.’s scheme, *E* applies the transmitted messages between *S*_*j*_ and *U*_*i*_ to acquire the access of server *S*_*k*_. To meet this problem, destination of message is added to the login request message and authentication request message, such as *M*_2_ = *h*(*AID*_*i*_||*N*_1_||*RPW*_*i*_||*SID*_*j*_||*T*_*i*_) and *M*_4_ = *h*(*SID*_*j*_||*N*_2_||*AID*_*i*_), so that *U*_*i*_ and *S*_*j*_ verify whether the one wants to be authenticated by the other one. At the same time, *E* cannot compute *M*_2_ or *M*_4_ without *N*_1_ or *N*_2_. Therefore, our scheme is secure against the masquerade attack.

#### Resistance to smart card attack

In the smart card attack, adversary *E* tries to apply the information obtained from smart card *SC*_*i*_ to be authenticated by server *S*_*j*_ without the password or biometrics. With SPA or DPA, *E* obtains *B*_*i*_, *C*_*i*_, *D*_*i*_ and *V*_*i*_ which are stored in *SC*_*i*_. In the proposed scheme, a session key between user *U*_*i*_ and server *S*_*j*_ is generated as follow.

$$\begin{array}{c} A_{i}=D_{i}⊕PSK⊕h\left(PSK\right), \end{array}$$

$$\begin{array}{c} N_{1}=RPW_{i}⊕M_{1}⊕h\left(PSK\right), \end{array}$$

$$\begin{array}{c} N_{2}=M_{3}⊕h(AID_{i}\left|\right|N_{1})⊕h\left(PSK\right), \end{array}$$

$$\begin{array}{c} SK_{ij}=h(AID_{i}||SID_{j}\left|\right|N_{1}\left|\right|N_{2}). \end{array}$$

Although *E* obtains *M*_1_ and *M*_3_ via public channels, it is difficult for him to retrieve *N*_1_, *N*_2_ and *AID*_*i*_ without *PSK*. Above all, our scheme prevents the smart card attack.

#### Resistance to user impersonation attack

The user impersonation attack means that adversary *E* impersonates user *U*_*i*_ using only smart card *SC*_*i*_ but without the password or biometrics. The proposed scheme applies *h*(*PSK*) to protect *N*_1_, *N*_2_ and *AID*_*i*_ even if *E* acquires *B*_*i*_, *C*_*i*_, *D*_*i*_ and *V*_*i*_ by side channel attacks. Thus, *E* cannot calculate the session keys to impersonate the user *U*_*i*_. In conclusion, our scheme resists the user impersonation attack.

#### Resistance to DoS attack

The DoS attack diminishes or eliminates the server’s expected capability to make the server unavailable. With the help of timestamp *T*_*i*_, server *S*_*j*_ checks the freshness and legality of *M*_2_ = *h*(*AID*_*i*_||*N*_1_||*RPW*_*i*_||*SID*_*j*_||*T*_*i*_) in the login request message. The current timestamp does not match the previous *M*_2_ which is sent by adversary *E*. Moreover, our scheme applies the fuzzy extractor to satisfy the usage requirements of biometrics. As a result, our scheme is secure against the DoS attack.

#### Resistance to server spoofing attack

Upon receiving the login request message from *U*_*i*_, adversary *E* tries to spoof as server *S*_*j*_ by replaying the old authentication request message $\left\{SID_{j},M_{3}^{old},M_{4}^{old}\right\}$, where $M_{3}^{old}=N_{2}^{old}⊕h(AID_{i}^{old}\left|\right|N_{1}^{old})⊕h\left(PSK\right)$ and $M_{4}^{old}=h(SID_{j}\left|\right|N_{2}^{old}||AID_{i}^{old})$. This attempt fails, since *U*_*i*_ uses different random numbers during different sessions, that is, $N_{1}^{old}\neq N_{1}^{new}$. Furthermore, *E* cannot acquire *RPW*_*i*_ to retrieve *N*_1_ from *N*_1_ = *RPW*_*i*_ ⊕ *M*_1_ ⊕ *h*(*PSK*). Therefore, our scheme prevents the server spoofing attack.

### Formal security analysis

With the help of the formal security analysis, we demonstrate that our scheme is secure against adversary *E*. For this purpose, we define oracle *Reveal* as follows: it unconditionally outputs *x* from one-way hash function *y* = *h*(*x*). The following two theorems provide the formal security analysis for our scheme.

**Theorem 1**. Under the assumption that one-way hash function *h*(⋅) closely behaves like oracle *Reveal*, our scheme is provably secure against adversary *E* for retrieving the identity *ID*_*i*_ of user *U*_*i*_, pre shared key *PSK* of server *S*_*j*_, and session key *SK*_*ij*_ between *U*_*i*_ and *S*_*j*_.

**Proof**. We need to construct adversary *E* who has the capacity to retrieve the identity *ID*_*i*_ of user *U*_*i*_, pre shared key *PSK* of server *S*_*j*_, and session key *SK*_*ij*_ between *U*_*i*_ and *S*_*j*_. Adversary *E* applies the oracle *Reveal* to execute the experimental algorithm $EXP1_{E,BMAKAS}^{HASH}$, where the BMAKAS means proposed biometric-based multi-server authentication and key agreement scheme. The details of Algorithm 1 are described in the Table 3.

**Table 3:** Algorithm $EXP1_{E,BMAKAS}^{HASH}$.

1. Eavesdrop the login request message {*AID*_*i*_, *M*_1_, *M*_2_, *B*_*i*_, *D*_*i*_, *T*_*i*_} during the login phase, where *AID*_*i*_ = *ID*_*i*_ ⊕ *h*(*N*_1_), *M*_1_ = *RPW*_*i*_ ⊕ *N*_1_ ⊕ *h*(*PSK*) and *M*_2_ = *h*(*AID*_*i*_||*N*_1_||*RPW*_*i*_||*SID*_*j*_||*T*_*i*_).
2. Apply the oracle *Reveal* to retrieve $AID_{i}^{I}$, $N_{1}^{I}$, $RPW_{i}^{I}$, $SID_{j}^{I}$ and $T_{i}^{I}$ from $Reveal\left(M_{2}\right)\to (AID_{i}^{I}\left|\right|N_{1}^{I}||RPW_{i}^{I}||SID_{j}^{I}\left|\right|T_{i}^{I})$.
3. **if** ($AID_{i}^{I}=AID_{i}$) **then**
4. Calculate $ID_{i}^{I}=AID_{i}^{I}⊕h\left(N_{1}^{I}\right)$ and $H_{1}=RPW_{i}^{I}⊕N_{1}^{I}⊕M_{1}$.
5. Apply the oracle *Reveal* to retrieve *PSK*^*I*^ from *Reveal*(*H*_1_) → (*PSK*^*I*^).
6. Eavesdrop the authentication request message {*SID*_*j*_, *M*_3_, *M*_4_} during the authentication phase, where *M*_3_ = *N*_2_ ⊕ *h*(*AID*_*i*_||*N*_1_) ⊕ *h*(*PSK*) and *M*_4_ = *h*(*SID*_*j*_||*N*_2_||*AID*_*i*_).
7. Further apply the oracle *Reveal* to retrieve $AID_{i}^{II}$, $N_{2}^{II}$ and $SID_{j}^{II}$ from $Reveal\left(M_{4}\right)\to (AID_{i}^{II}\left|\right|N_{2}^{II}||SID_{j}^{II})$.
8. **if** ($SID_{j}=SID_{j}^{II}$) and $\left(AID_{i}=AID_{i}^{II}\right)$ **then**
9. Calculate $H_{2}=N_{2}^{II}⊕h(AID_{i}^{I}\left|\right|N_{1}^{I})⊕M_{3}$.
10. Apply the oracle *Reveal* to retrieve *PSK*^*II*^ from *Reveal*(*H*_2_) → (*PSK*^*II*^).
11. **if** (*PSK*^*I*^ = *PSK*^*II*^) **then**
12. Calculate $SK_{ij}^{*}=h(AID_{i}||SID_{j}\left|\right|N_{1}^{I}\left|\right|N_{2}^{II})$.
13. Accept $ID_{i}^{I}$, *PSK*^*I*^ and $SK_{ij}^{*}$ as the identity *ID*_*i*_ of user *U*_*i*_, pre shared key *PSK* of server *S*_*j*_, and session key *SK*_*ij*_ between *U*_*i*_ and *S*_*j*_, respectively.
14. **return** 1 (Success)
15. **else**
16. **return** 0 (Failure)
17. **end if**
18. **else**
19. **return** 0 (Failure)
20. **end if**
21. **else**
22. **return** 0 (Failure)
23. **end if**

And we define the success probability of $EXP1_{E,BMAKAS}^{HASH}$ as $Success1=|P(EXP1_{E,BMAKAS}^{HASH}=1)-1|$, where *P*(⋅) means the probability of $EXP1_{E,BMAKAS}^{HASH}$. The advantage function for algorithm $EXP1_{E,BMAKAS}^{HASH}$ becomes *Adv*1(*et*_1_, *q*_*Reveal*_) = max{*Success*1}, where the maximum for adversary *E* depends on the execution time *et*_1_ and number of queries *q*_*Reveal*_ made to the oracle *Reveal*. Our scheme is provably secure against adversary *E*, if *Adv*1(*et*_1_, *q*_*Reveal*_) ≤ *ε*_1_, for any sufficiently small *ε*_1_ > 0. If adversary *E* has the ability to retrieve *x* from one-way hash function *y* = *h*(*x*), then he can easily derive the identity *ID*_*i*_, pre shared key *PSK* and session key *SK*_*ij*_ to win the game. However, it is a computationally infeasible problem to retrieve the inputs of one-way hash function. So max_*E*_{*Success*1} = *Adv*1(*et*_1_, *q*_*Reveal*_) ≤ *ε*_1_, for any sufficiently small *ε*_1_ > 0. In conclusion, our scheme is provably secure against adversary *E* for retrieving the identity *ID*_*i*_ of user *U*_*i*_, pre shared key *PSK* of server *S*_*j*_, and session key *SK*_*ij*_ between *U*_*i*_ and *S*_*j*_.

**Theorem 2**. Under the assumption that one-way hash function *h*(⋅) closely behaves like oracle *Reveal*, our scheme is provably secure against adversary *E* for retrieving the password *PW*_*i*_ of user *U*_*i*_, even if smart card *SC*_*i*_ is stolen.

**Proof**. We need to construct the adversary *E* who has the capacity to retrieve the password *PW*_*i*_. Adversary *E* extracts all the information {*B*_*i*_, *C*_*i*_, *D*_*i*_, *V*_*i*_} from stolen smart card *SC*_*i*_ and applies the oracle *Reveal* to execute the experimental algorithm $EXP2_{E,BMAKAS}^{HASH}$. The details of Algorithm 2 are described in the Table 4.

**Table 4:** Algorithm $EXP2_{E,BMAKAS}^{HASH}$.

1. Extract all the information {*B*_*i*_, *C*_*i*_, *D*_*i*_, *V*_*i*_} from stolen smart card *SC*_*i*_ with the help of side channel attacks, where *V*_*i*_ = *h*(*ID*_*i*_||*RPW*_*i*_) and *RPW*_*i*_ = *h*(*PW*_*i*_||*R*_*i*_).
2. Apply the oracle *Reveal* to retrieve $ID_{i}^{I}$ and $RPW_{i}^{I}$ from $Reveal\left(V_{i}\right)\to (ID_{i}^{I}||RPW_{i}^{I})$.
3. Eavesdrop the login request message {*AID*_*i*_, *M*_1_, *M*_2_, *B*_*i*_, *D*_*i*_, *T*_*i*_} during the login phase, where *AID*_*i*_ = *ID*_*i*_ ⊕ *h*(*N*_1_) and *M*_2_ = *h*(*AID*_*i*_||*N*_1_||*RPW*_*i*_||*SID*_*j*_||*T*_*i*_).
4. Apply the oracle *Reveal* to retrieve $AID_{i}^{II}$, $N_{1}^{II}$, $RPW_{i}^{II}$, $SID_{j}^{II}$ and $T_{i}^{II}$ from $Reveal\left(M_{2}\right)\to (AID_{i}^{II}\left|\right|N_{1}^{II}||RPW_{i}^{II}||SID_{j}^{II}\left|\right|T_{i}^{II})$.
5. Calculate $ID_{i}^{II}=AID_{i}^{II}⊕h\left(N_{1}^{II}\right)$.
6. **if** ($ID_{i}^{I}=ID_{i}^{II}$) **then**
7. Apply the oracle *Reveal* to retrieve $PW_{i}^{I}$ and $R_{i}^{I}$ from $Reveal\left(RPW_{i}^{I}\right)\to (PW_{i}^{I}\left|\right|R_{i}^{I})$.
8. Accept $PW_{i}^{I}$ as the password *PW*_*i*_ of user *U*_*i*_.
9. **return** 1 (Success)
10. **else**
11. **return** 0 (Failure)
12. **end if**

Also we define the success probability of $EXP2_{E,BMAKAS}^{HASH}$ as $Success2=|P(EXP2_{E,BMAKAS}^{HASH}=1)-1|$, where *P*(⋅) means the probability of $EXP2_{E,BMAKAS}^{HASH}$. The advantage function for algorithm $EXP2_{E,BMAKAS}^{HASH}$ becomes *Adv*2(*et*_2_, *q*_*Reveal*_) = max_*E*_{*Success*2}, where the maximum for adversary *E* depends on the execution time *et*_2_ and number of queries *q*_*Reveal*_ made to the oracle *Reveal*. Our scheme is provably secure against adversary *E*, if *Adv*2(*et*_2_, *q*_*Reveal*_) ≤ *ε*_2_, for any sufficiently small *ε*_2_ > 0. If adversary *E* has the ability to retrieve *x* from one-way hash function *y* = *h*(*x*), then he can easily derive the password *PW*_*i*_ to win the game. However, it is a computationally infeasible problem to retrieve the inputs of one-way hash function. So max_*E*_{*Success*2} = *Adv*2(*et*_2_, *q*_*Reveal*_) ≤ *ε*_2_, for any sufficiently small *ε*_2_ > 0. In conclusion, our scheme is provably secure against adversary *E* for retrieving the password *PW*_*i*_ of user *U*_*i*_.

### Functionality analysis

Various functionality requirements for a multi-server authentication and key agreement scheme have been suggested in previous studies. In this section, we show that our scheme provides these functionalities.

#### Anonymity

The anonymity means that user’s real identity is not disclosed to an unauthorized party. In the presented scheme, *U*_*i*_ calculate the dynamic identity *AID*_*i*_ from *AID*_*i*_ = *ID*_*i*_ ⊕ *h*(*N*_1_), and *N*_1_ does not leak out from the messages over public channels. Thus, adversary *E* cannot compute the user’s identity *ID*_*i*_ without *N*_1_. The authorized server *S*_*j*_ retrieves *A*_*i*_ = *D*_*i*_ ⊕ *PSK* ⊕ *h*(*PSK*) and *RPW*_*i*_ = *B*_*i*_ ⊕ *h*(*A*_*i*_), and further calculates *N*_1_ from *N*_1_ = *RPW*_*i*_ ⊕ *M*_1_ ⊕ *h*(*PSK*). So only authorized servers confirm the real identity of *U*_*i*_. As a result, adversary *E* cannot acquire the user’s real identity, but user *U*_*i*_ is authenticated anonymously by server *S*_*j*_.

#### Mutual authentication

The mutual authentication is achieved when two parties authenticate each other. In our scheme, users and servers authenticate each other by using *N*_1_, *N*_2_, *h*(*PSK*), *D*_*i*_ and *T*_*i*_. During the authentication phase, server *S*_*j*_ verifies whether *M*_2_ is consistent with *h*(*AID*_*i*_||*N*_1_||*RPW*_*i*_||*SID*_*j*_||*T*_*i*_) to authenticate the user *U*_*i*_. And *U*_*i*_ authenticates *S*_*j*_ by checking whether *h*(*SID*_*j*_||*N*_2_||*AID*_*i*_) = *M*_4_ holds. In conclusion, our scheme provides the mutual authentication.

#### Session key agreement

The session key agreement means that users and servers securely establish a session key which is applied for protecting the subsequent communication. In the proposed scheme, a session key *SK*_*ij*_ = *h*(*AID*_*i*_||*SID*_*j*_||*N*_1_||*N*_2_) is generated by user *U*_*i*_ and server *S*_*j*_, where *N*_1_ and *N*_2_ are different in every session. Therefore, session keys are different in each session so that it is difficult for adversary *E* to retrieve the previous session keys from the intercepted messages.

#### Perfect forward secrecy

The perfect forward secrecy means that a session key will not be compromised if the user’s long-term key is compromised in the future [11, 15]. In our scheme, a session key between user *U*_*i*_ and server *S*_*j*_ is calculated as follow.

$$\begin{array}{c} A_{i}=D_{i}⊕PSK⊕h\left(PSK\right), \end{array}$$

$$\begin{array}{c} RPW_{i}=B_{i}⊕h\left(A_{i}\right), \end{array}$$

$$\begin{array}{c} N_{1}=RPW_{i}⊕M_{1}⊕h\left(PSK\right), \end{array}$$

$$\begin{array}{c} N_{2}=M_{3}⊕h(AID_{i}\left|\right|N_{1})⊕h\left(PSK\right), \end{array}$$

$$\begin{array}{c} SK_{ij}=h(AID_{i}||SID_{j}\left|\right|N_{1}\left|\right|N_{2}). \end{array}$$

Although user’s long-term key *h*(*PSK*) is compromised, adversary *E* cannot calculate *RPW*_*i*_ and *PSK* so that he cannot retrieve *N*_1_ and *N*_2_ to generate the session keys between *U*_*i*_ and *S*_*j*_. Above all, our scheme achieves the perfect forward secrecy.

#### User revocation/re-registration

The user *U*_*i*_ needs to send a revocation or re-registration request message to the registration center *RC* over a secure channel if he wants to revoke his privilege or re-register. *RC* help *U*_*i*_ revoke his privilege or re-register by modifying 〈*ID*_*i*_, *N*_*i*_〉 in the database. The functionality of user revocation/re-registration meets the requirements of practical applications. It also makes our scheme more robust than other related schemes.

#### Biometric information protection

In conventional scheme, biometric information of user is directly stored in the smart card *SC*_*i*_ so that adversary *E* obtains biometrics from lost smart card with the assistance of side channel attacks. We adopt a high security mechanism to solve this problem. The nearly random string *R*_*i*_ is protected by one-way hash function, which is extracted from biometric information *BIO*_*i*_ by fuzzy extractor. And more details are described in Section 2.2. So it makes impossible for *E* to obtain the biometric information. In conclusion, our scheme provides the biometric information protection.

### Efficiency analysis

The efficiency is an important consideration in the aspect of evaluating the schemes. The efficiency of a multi-server authentication and key agreement scheme can be measured by the following metrics, single registration, secure and simple password modification, fast error detection, and low computational cost.

#### Single registration

The single registration means that a single point of registration allows users to acquire the access to all servers in the system. In the proposed scheme, user *U*_*i*_ registers with registration center *RC* only once to be authenticated with every server and apply the server’s services anonymously. So our scheme achieves the single registration.

#### Secure and simple password modification

The secure and simple password modification demands that users change their passwords without the assistance of any third trusted party and the authenticity of the users is verified by their smart card. In our scheme, user *U*_*i*_ changes the password conveniently and does not require any communication with registration center *RC*. Furthermore, smart card *SC*_*i*_ checks whether *h*(*ID*_*i*_||*RPW*_*i*_) = *V*_*i*_ holds for every password modification so that adversary *E* cannot change the password even if he acquires the smart card and password. In conclusion, proposed scheme provides the secure and simple password modification.

#### Fast error detection

It is necessary to provide the fast error detection, which means that smart card *SC*_*i*_ checks the incorrect passwords or any other discrepancies quickly. In the login and password change phases, *SC*_*i*_ detects the errors immediately, such as inaccurate identities, incorrect passwords and false biometrics without the help of registration center *RC* and server *S*_*j*_. Therefore, our scheme achieves the fast error detection.

#### Low computational cost

The computational cost of the scheme should be minimized in practice. As the major parties of communication, *U*_*i*_ and *S*_*j*_ produce the random number twice, calculate the XOR operation 12 times, and perform the hash function 15 times to complete the login and authentication phases. As a result, computational cost of our scheme is a little lower than other related schemes.

### Comparisons with related schemes

In this section, we compare the resistance, functionality and performance of our scheme with other related existing biometric-based multi-server authentication and key agreement schemes, such as Chuang et al.’s scheme [51], Mishra et al.’s scheme [53], Xue et al.’s scheme [59] and Li et al.’s scheme [60].

Table 5 lists the resistance comparison of various biometric-based multi-sever authenticated key agreement schemes. We define the following notations: R1: resistance to replay attack, R2: resistance to modification attack, R3: resistance to stolen-verifier attack, R4: resistance to off-line guessing attack, R5: resistance to forgery attack, R6: resistance to insider attack, R7: resistance to masquerade attack, R8: resistance to smart card attack, R9: resistance to user impersonation attack, R10: resistance to DoS attack and R11: resistance to server spoofing attack in the Table 5. The result indicates that our scheme is more secure and achieves the all resistance requirements.

**Table 5:** The resistance comparison.

	Chuang et al.’s [51]	Mishra et al.’s [53]	Xue et al.’s [59]	Li et al.’s [60]	Ours
R1	No	No	No	No	Yes
R2	Yes	Yes	Yes	Yes	Yes
R3	Yes	Yes	No	No	Yes
R4	Yes	Yes	No	No	Yes
R5	Yes	Yes	Yes	Yes	Yes
R6	Yes	Yes	No	Yes	Yes
R7	No	No	No	No	Yes
R8	No	Yes	Yes	No	Yes
R9	No	Yes	Yes	Yes	Yes
R10	No	No	Yes	Yes	Yes
R11	Yes	Yes	No	Yes	Yes

Table 6 shows the functionality comparison of proposed scheme with other related schemes. In the Table 6, we use the following notations: F1: anonymity, F2: mutual authentication, F3: session key agreement, F4: perfect forward secrecy, F5: user revocation/re-registration and F6: biometric information protection. And we further compare our scheme with Lu et al.’s scheme [24] which is another improved scheme. It can be seen that our scheme provides more functionality requirements than other related schemes.

**Table 6:** The functionality comparison.

	Chuang et al.’s [51]	Mishra et al.’s [53]	Xue et al.’s [59]	Li et al.’s [60]	Lu et al.’s [48]	Ours
F1	Yes	Yes	Yes	Yes	Yes	Yes
F2	No	Yes	Yes	Yes	Yes	Yes
F3	Yes	Yes	Yes	Yes	Yes	Yes
F4	No	No	Yes	Yes	Yes	Yes
F5	No	No	No	No	No	Yes
F6	No	Yes	No	No	Yes	Yes

**Fig 8:**
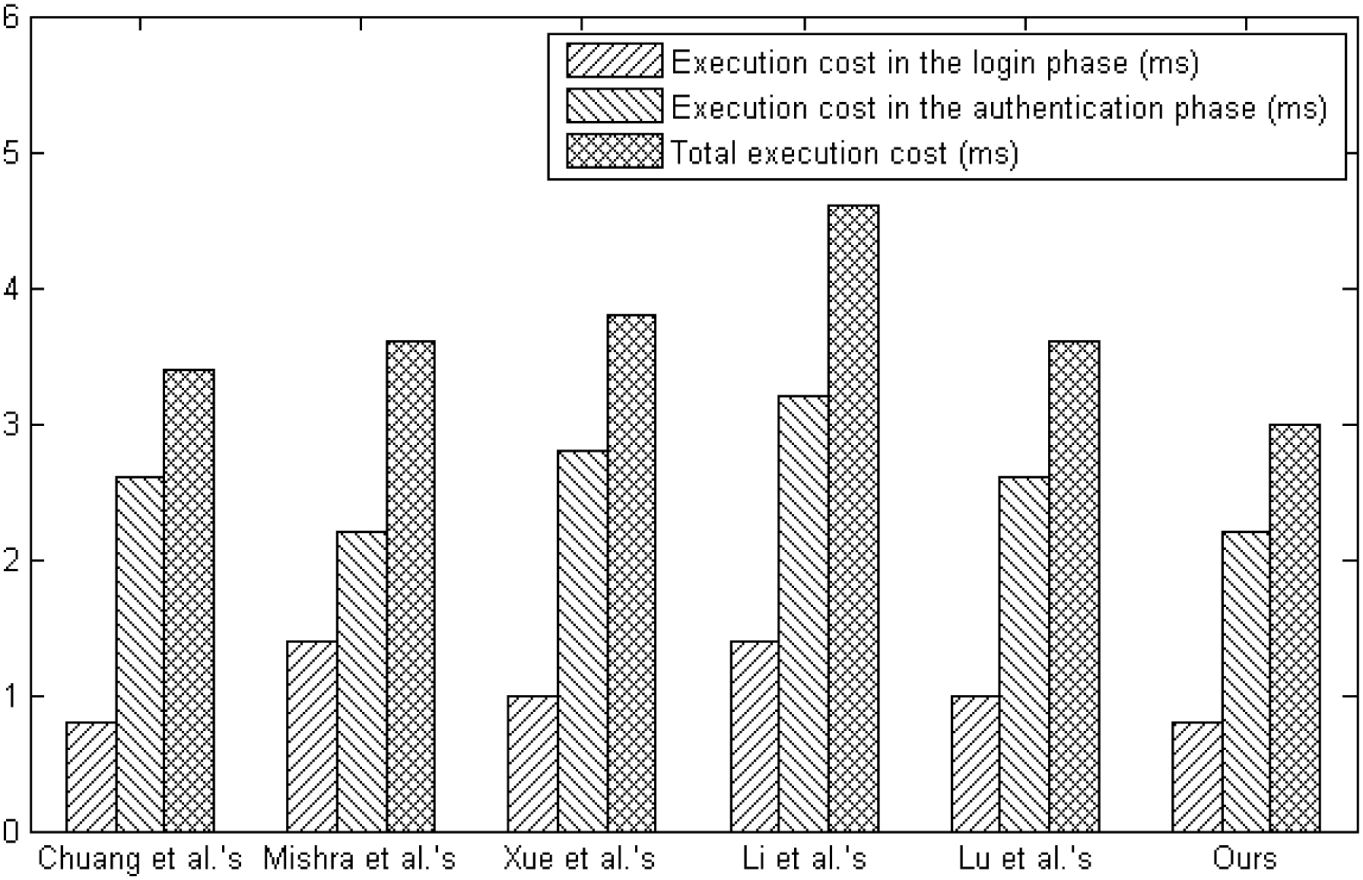
The computation cost comparison.

We compare our scheme with other biometric-based multi-sever authentication and key agreement schemes for computational overhead, communication overhead and storage requirement involved in the login and authentication phases. In order to measure the computational complexity, we apply the number of hash function operations as time complexity since the XOR operation requires very little computational cost, where *T*_*h*_ stands for the computation time for hash function. According to the Xue et al.’s work [61], we learn that the average running time of a one-way secure hash function operation is about 0.2 ms. As shown in the Table 7 and Fig 8, we demonstrate the comparison among our scheme and other related schemes in terms of the computation overhead. In the Table 7, we use the following notations: S1: computation overhead in the login phase, S2: execution overhead in the login phase, S3: computation overhead in the authentication phase, S4: execution overhead in the authentication phase and S5: total execution overhead. The proposed scheme requires lower computation overhead than other schemes.

**Table 7:** The computation cost comparison.

	Chuang et al.’s [51]	Mishra et al.’s [53]	Xue et al.’s [59]	Li et al.’s [60]	Lu et al.’s [48]	Ours
S1	4*T*_*h*_	7*T*_*h*_	5*T*_*h*_	7*T*_*h*_	4*T*_*h*_	4*T*_*h*_
S2	0.8ms	1.4ms	1.0ms	1.4ms	1.0ms	0.8ms
S3	13*T*_*h*_	11*T*_*h*_	14*T*_*h*_	16*T*_*h*_	13*T*_*h*_	11*T*_*h*_
S4	2.6ms	2.2ms	2.8ms	3.2ms	2.6ms	2.2ms
S5	3.4ms	3.6ms	3.8ms	4.6ms	3.6ms	3.0ms

**Fig 9:**
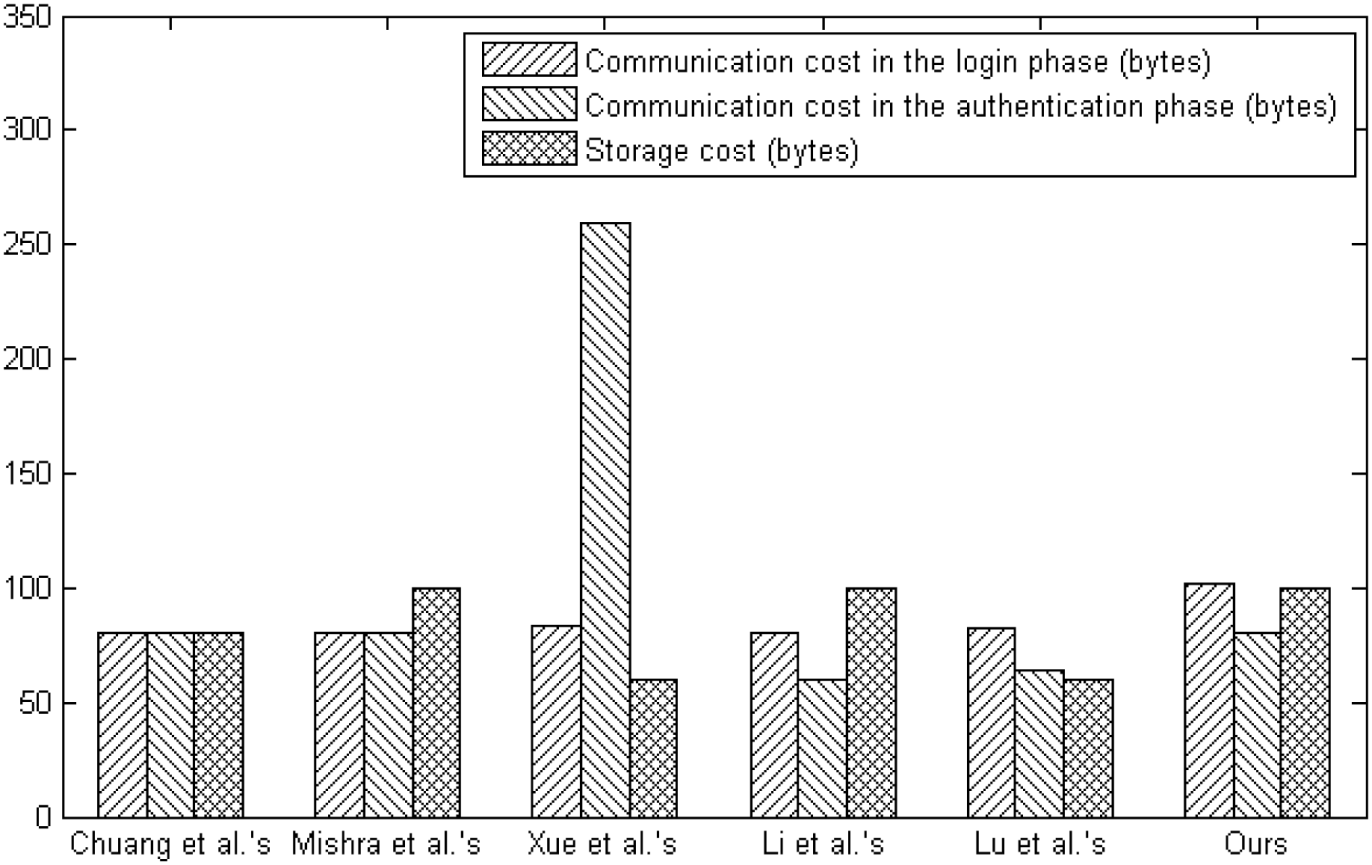
The communication and storage costs comparison.

To estimate the communication efficiency, we assume that the length of security parameters, such as the bit length of random number *N*_*i*_ is 160, the bit length of user identity is 160, the bit length of timestamp *T*_*i*_ is 16 and the output length of hash function is 160 if we follow the SHA-1 which is applied in the most of previous schemes. In our scheme, *U*_*i*_ transmits the request message {*AID*_*i*_, *M*_1_, *M*_2_, *B*_*i*_, *D*_*i*_, *T*_*i*_} to *S*_*j*_ during the login phase, and its length is (160 + 160 + 160 + 160 + 160 + 16)/8 = 102bytes. And in the stage of authentication, communication overhead is (160 + 160 + 160 + 160)/8 = 80bytes, which contains the authentication request message {*SID*_*j*_, *M*_3_, *M*_4_} and authentication reply {*M*_5_}. So total communication overhead of proposed scheme is 102 + 80 = 182bytes. Analogously, we measure the communication overhead of related schemes. In order to estimate the storage requirement, we consider the messages stored in the smart card as the storage overhead and calculate the byte length of stored information. In our scheme, the stored message {*B*_*i*_, *C*_*i*_, *D*_*i*_, *V*_*i*_, *P*_*i*_,} requires (160 + 160 + 160 + 160 + 160)/8 = 100bytes. Similarly, we estimate the storage requirement of other schemes. Table 8 and Fig 9 show the comparisons regarding on the communication and storage costs of various multi-sever authentication and key agreement schemes. We provide the following notations: C1: communication cost in the login phase, C2: communication cost in the authentication phase, C3: total communication cost and C4: storage cost in the Table 8. With the same level of communication overhead and storage requirement, our scheme obviously has advantages in the computational complexity by considering the computation cost of these related schemes. From the results of comparisons given above, we conclude that our scheme has better efficiency between resistance, functionality and performance than other related schemes.

**Table 8:** The communication and storage costs comparison.

	Chuang et al.’s [51]	Mishra et al.’s [53]	Xue et al.’s [59]	Li et al.’s [60]	Lu et al.’s [48]	Ours
C1	80bytes	80bytes	83bytes	80bytes	82bytes	102bytes
C2	80bytes	80bytes	259bytes	60bytes	64bytes	80bytes
C3	160bytes	160bytes	342bytes	140bytes	146bytes	182bytes
C4	80bytes	100bytes	60bytes	100bytes	60bytes	100bytes

## Conclusion

With the security requirements of networks, biometrics authenticated schemes which are applied in the multi-server environment come to be more crucial and widely deployed. In this paper, we analyze the security of Mishra et al.’s scheme. Based on the cryptanalysis of their scheme, we propose a novel biometric-based multi-server authentication and key agreement scheme. The presented scheme improves the Mishra et al.’s scheme, and satisfies the desirable security requirements which are demonstrated through informal and formal security analysis respectively. Also our scheme provides some significant functionalities which are not considered in the most of existing authentication schemes, such as, user revocation or re-registration and biometric information protection. In addition, comparisons in the security, functionality and performance between proposed scheme and several related ones are given. The results show that our scheme has more secure properties, more functionalities and lower computation cost with the same level of communication overhead and storage requirement. We conclude that our scheme is obviously more appropriate for practical applications in the remote distributed networks.
